# A Research Based on Online Medical Platform: The Influence of Strong and Weak Ties Information on Patients’ Consultation Behavior

**DOI:** 10.3390/healthcare10060977

**Published:** 2022-05-24

**Authors:** Yuting Zhang, Chutong Qiu, Jiantong Zhang

**Affiliations:** 1 School of Economics and Management, Tongji University, Shanghai 200092, China; z_y_t2019@tongji.edu.cn; 2 Courant Institute of Mathematical Sciences, New York University, New York, NY 10012, USA; cq2039@nyu.edu

**Keywords:** patients’ consultation behavior, strong ties, weak ties, online medical platform

## Abstract

As an indispensable part of contemporary medical services, Internet-based medical platforms can provide patients with a full range of multi-disciplinary and multi-modal treatment services. Along with the emergence of many healthcare influencers and the increasing connection between online and offline consultations, the operation of individual physicians and their teams on Internet-based medical platforms has started to attract a lot of attention. The purpose of this paper is to, based on an Internet platform, study how the information on physicians’ homepages influences patients’ consultation behavior, so as to provide suggestions for the construction of physicians’ personal websites. We distinguish variables into strong- and weak-ties types, dependent on whether deep social interactions between physicians and patients have happened. If there exist further social interactions, we define the variable as the “strong ties” type, otherwise, “weak ties”. The patients’ consultation behavior will be expressed as the volume of online consultation, i.e., the number of patients. We obtained the strong and weak ties information of each physician based on EWM (entropy weight method), so as to establish a regression model with explained variable, i.e., the number of patients, and three explanatory variables, i.e., the strong and weak ties information, and their interaction term. The estimation results verified our hypotheses and proved to be robust. It showed that both strong and weak ties information can positively influence patients’ consultation behavior, and the influence of weak ties information is greater. Regarding the positive influence of strong and weak ties, we found a trade off effect between them. Based on the results, we finalize with some suggestions on how to improve a physician’s online medical consultation volume.

## 1. Introduction

Online healthcare consultation has become an essential part of healthcare system. Online medical platforms provide patients with a channel that allows them to make an appointment, learn about a physician, understand their severity of illness, and ask for advice on the Internet without having to leave home [1,2,3].

Researchers focus on different kinds of information in the online health community to investigate how factors impact patients’ consultation behavior. The Information of physicians contains the self-disclosed information, online image, etc. [1,2,4,5]. In addition, consumer value was co-created by the online medical community [6].

The current physician–patient relationship in China is experiencing dilemmas, which are partly due to the information asymmetry [7,8,9] between physicians and patients. A physician’s personal homepage can provide a channel for patients to learn about physicians and diseases, thus providing a service for patients to choose a physician based on detailed information [10,11], which, to some extent, alleviates information asymmetry and also exerts an influence on patients’ consultation behavior [12,13]. The literature has divided physician homepage information into system-generated and patient-generated information. For example, thank-you notes are data generated after a patient consultation and are classified as patient-generated information, while contribution values, etc., are treated as system-generated information [14,15]. It is generally agreed that positive physician services information of both kinds of information can positively affect patient consultations. Patients with different diseases and different types of interest can all use the information generated by the system, such as online reputation, online workload, etc., to make a comprehensive assessment of the physician and make a physician selection decision. They can also view patient-generated information, such as past physician–patient interactions. Both patient-generated information and system-generated information help patients to make decisions [14]. The research on patient-generated health data becomes popular, especially because of the rise of social media [16], and these data are used in multiple fields [17]. It has been well documented that the interaction between physicians and patients also influences patients’ consultation behavior [18,19,20,21]. The researcher analyzed the impact of reputation from multi and cross-level perspectives [22]. Patient-generated information, which is regarded as a wealth of “big data” [23], also helps evaluate health interest [24], which is important to analyze the doctor–patient relationship [20]. In addition, in the patients’ consultation behavior, these two types of information can have an impact on each other’s roles. For example, the physician’s word of mouth on the Internet has a positive effect on the patient’s choice of consultation, while the risks and knowledge associated with the disease weakens the positive impact of electronic word of mouth on the patient [3,4].

The online medical platform and patients can essentially be regarded as an internet product and its users. So, the social relationship of this platform itself can objectively affect user behavior. The impact of online social relationship strength on user behavior is also an important research topic. Current research on social interaction suggests that people’s social relationships are related to the strength of their ties, which are defined as weak and strong ties. Weak ties between people are more important in the process of information dissemination than strong ties [25,26,27]. There is empirical research examining the role of strong and weak ties in civic online interaction. The strength of ties for civic engagement was found to be similar online or in reality, while ties among patients are regarded as weak [28]. For example, the strong professionalism of physicians and the anxiety caused by patients’ diseases are strongest, which are regarded as strong ties, in predicting civic behavior and play an important mediating role in online and offline communication, with weak ties appearing more online [29]. When extended to online social networking, the concept of social strength of interaction still holds true. In an analysis of two social media platforms, Facebook and Twitter, it was found that strong ties showed more effective communication on Facebook, while weak ties performed better on Twitter [30]. However, scholars have also rejected the hypothesis that weak relationships have a uniquely important supportive role in social networks, arguing that ‘strong relationship strengths’ provide more emotional and informational support and are more supported by participants from a psychological perspective [31].

On the other hand, patient behavior can also exert an influence on physicians. Whether or not patients choose to ask for a consultation is also used as an indicator to measure physician services, which will further motivate physicians to improve the quality of their services [32]. Therefore, this paper will provide some assistance to physicians based on the analysis of patient behavior.

In order to research the influence of physicians’ information on patients’ consultation behavior, we use physicians’ data from Online Medical Platform A, a well-known Chinese online medical platform. The data are classified into strong and weak ties information based on the social ties perspective, so as to investigate the impact of physician–patient interaction intensity on patients’ consultation behavior. We will explore the role of online medical information on patient behavior from a new perspective and will further improve the research about how online social relationship strength information influences user behavior in the online medical field. Furthermore, the results, to some extent, can provide the support of theoretical suggestions to solve physician–patient conflicts caused by the information gap between physicians and patients in the Internet medical field.

Our study will explore the answers to the following questions:

(1) How do strong and weak ties information affect patients’ consultation behavior through the online medical platform?
(2) Which has more influence on patients’ consultation behavior? Strong ties information or weak ties information?
(3) Do strong and weak ties exert a trade-off effect on each other’s influence on patients’ consultation behavior? For example, does the enhancement of weak ties information reduce the positive effect of strong ties information on the patients’ consultation behavior?
(4) The influence of non-social ties information, i.e., some nature of the physician himself, on the patients’ consultation behavior.

## 2. Methods

### 2.1. Data Sources

We used Python to crawl 52,645 physicians’ homepages on 18 January 2022 and 25 January 2022 from the well-known Chinese Online Medical Platform A for model building and robustness test. From the perspective of horizontal physicians’ homepages, each datum contains numerical and textual information that users can see when they visit physicians’ homepages. From the platform perspective, the 52,645 data we crawled account for 22% of the total number of registered physicians, which is a very large sample. Such a rich and large volume of data will help us to more fully explore the information behind the data and explore the impact of physicians’ homepage information on patients’ consultation behavior.

### 2.2. Variables

We select “Number of checked patients (*NCP*)”, “Number of comments after consultation (*NCC*)”, “Number of thank-you letters (*NTL*)”, “Number of gifts (*NOG*)”, and “Number of online patients (*NOP*)” to form strong ties. The variables such as “Comprehensive recommendation score (*CRS*)”, “Number of articles on Health subscription (*NAH*)”, and “Number of articles reads (*NAR*)” are used to form weak ties. These variables will be processed later to form three independent variables: *WeakTies*, *StrongTies*, and the interaction term *WeakStrongTies*, which will be the real independent variables needed for the models.

In this paper, we choose the number of patients who have consulted physicians as the dependent variable. This is the original collected variable that is automatically displayed on the physician’s homepage, and can indicate the number of online consultation that the physician has received. We use this variable to measure the patients’ consultation behavior. A higher number of patients indicates a more active patient consultation.

We also add *dummy* variables to the model. The title, education title, and outpatient information are set as *dummy* variables. We coded title *dummy* from 1 to 4, e.g., *title*_*dummy*1, coded education title *dummy* from 1 to 6, *edutitle*_*dummy*1. In order to measure the information of the highest level of outpatient consultation type that a physician can provide offline, we coded outpatient *dummy* from 1 to 8, e.g., *op*_*dummy*1.

Table 1 shows the definitions and descriptive statistics of all original variables.

### 2.3. Hypothesis Strong Ties Models and Hypothesis

To explore how information on physicians’ homepage impact patients’ consultation behavior, we distinguish the initial information into strong ties information and weak ties information based on whether deep social interactions between physicians and patients have happened. If there exist deep social interactions, we define these kinds of initial variables as “strong ties” variables; otherwise, we define those initial variables as “weak ties” variables.

#### 2.3.1. Strong Ties

In online medical platforms, patients and physicians will have close interactions, including but not limited to offline consultation, evaluation of physician’s service, following physicians, expressing gratitude through thank-you letters and gifts, and so on. Therefore, this paper selects the patient-generated information data, i.e., “Number of checked patients (*NCP*)”, “Number of comments after consultation (*NCC*)”, “Number of thank-you letters (*NTL*)”, “Number of gifts (*NOG*)”, and “Number of followers (*NOF*)”, generated by the in-depth interaction between the physician and the patient to form the “strong ties” behavior indicator. Thus, the following hypotheses are proposed:

**Hypothesis** **1.** *Strong ties have a positive effect on the patients’ consultation behavior [33]*.

#### 2.3.2. Weak Ties

System-generated information exists on the physician’s homepage, such as the number of reads of articles published on the physician’s website health subscription (*NAR*). Although some of these data, such as the comprehensive recommendation score rated by Online Medical Platform A, are jointly calculated by the algorithm based on some patient-generated information and system-generated information on the physician’s homepage. However, in general, this part of the data is mostly determined by the system, and the interaction between patients and physicians cannot exert too much influence on it. So, we use the explicit website data, i.e., “Comprehensive recommendation score (*CRS*)”, “Number of articles on Health subscription (*NAH*)”, and “Number of articles reads (*NAR*)”, to form “weak ties” behavior indicators. Thus, the following hypotheses are proposed:

**Hypothesis** **2.** *Weak ties have a positive effect on the patients’ consultation behavior.*

#### 2.3.3. Interaction of Weak Ties and Strong Ties

System-generated information and patient-generated information will have an effect on each other’s influence on the patients’ consultation behavior, so we plan to investigate the interaction of strong and weak ties. Thus, we propose the following hypothesis:

**Hypothesis** **3.** *The enhancement of weak ties information will reduce the positive effect of strong ties information on the patients’ consultation behavior [34]*.

**Figure 1:**
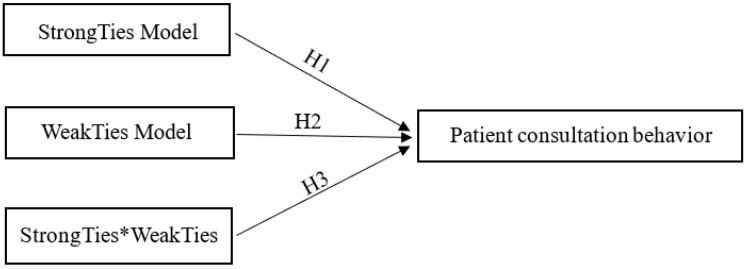
Hypothetical model of influencing factors of patients’ consultation behavior.

Figure 1 shows the hypothetical model of influencing factors of patients’ consultation.

### 2.4. Data

#### 2.4.1. Data Pre-Processing

When the total number of visits or total number of patients of a physician is too small, there will be a shortage of multiple data for this physician, so his or her data are not referenceable. Therefore, physicians with total visits less than 1000 were considered as abnormal data and were deleted. Finally, 42,319 pieces of data were retained.

After data cleaning, Python’s sklearn.preprocessing package was used to scale the values and take the logarithm of the factor patients, thus completing the normalization of the data.

#### 2.4.2. Strong and Weak Ties Model

In the process of constructing the strong and weak ties model, considering that the information performance of the original variables is fairly objective, we did not choose the subjective weighting method. Instead, we chose the objective weighting entropy method to assign weights to the original indicators and complete the classification calculation based on the coefficients, so as to build the required strong and weak ties model:

(1) Strong Ties Model:

(1) $$StrongTies_{i}=X_{1}NCP_{i}+X_{2}NCC_{i}+X_{3}NTL_{i}+X_{4}NOG_{i}+X_{5}NOF_{i}$$

(2) Weak Ties Model:

(2) $$WeakTies_{i}=Y_{1}CRS_{i}+Y_{2}NAH_{i}+Y_{3}NAR_{i}$$

(3) Interaction Term of Weak Ties and Strong Ties:

(3) $$StrongWeakTies_{i}=StrongTies_{i}\times StrongTies_{i}$$

We used Python to calculate the entropy method assignment coefficients and bring them into the Formulas (1) and (2), respectively, to obtain the following models:

(4) Strong Ties Model:

(4) $$StrongTies_{i}=0.227NCP_{i}+0.168NCC_{i}+0.191NTL_{i}+0.224NOG_{i}+0.190NOF_{i}$$

(5) Weak Ties Model:

(5) $$WeakTies_{i}=0.005CRS_{i}+0.527NAH_{i}+0.468NAR_{i}$$

Finally, we obtained the data of three variables, *StrongTies_i_*, *WeakTies_i_*, and *StrongWeakTies_i_*. We used these three as partial independent variables and participated in the construction of the regression model.

Table 2 shows the definitions of the Variables and summary statistics, and Table 3 presents the correlations of the Variables. The variables “*op*-dummy*” are the *dummy* variables generated by the variable “outpatient”, which represents the highest level of outpatient consultation type that a physician can provide offline. As shown in Table 2, e.g., “*op*_*dummy*1” represents the number of physicians whose highest level of outpatient consultation type is “VIP5-VIP outpatient”.

### 2.5. Regression Model

We construct models 1–5 separately using ordinary least squares (OLS) regression.

#### 2.5.1. Model 1

Since the number of consultations on online medical platforms represents the attractiveness of physicians to patients, we constructed five models to test how two types of information, weak ties and strong ties information, impact patients’ consultation behavior. Model 1, which is our baseline model, is configured as follows:

(6) $$\ln Patients_{i}=\alpha_{0}+\alpha_{1}WeakTies_{i}+\alpha_{2}StrongTies_{i}+\alpha_{3}\left(WeakTies_{i}\times StrongTies_{i}\right)+\varepsilon_{i}$$

In this model, *i* = 1, 2, …, 5 presents physicians’ id numbers. The dependent variable “*Patients_i_*” is the total number of patients who have consulted physicians. Variable “*StrongTies_i_*” is generated by Equation (1), and variable “*WeakTies_i_*” is generated by Equation (2).

#### 2.5.2. Model 2

Considering that some confounding factors may have an influence on the relationship between social ties information and patients’ consultation behavior, we chose physicians’ title information as a *dummy* variable to join the model:

(7) $$\begin{array}{ll} \ln Patients_{i} & =\alpha_{0}+\alpha_{1}WeakTies_{i}+\alpha_{2}StrongTies_{i}+\alpha_{3}\left(WeakTies_{i}\times StrongTies_{i}\right)+\beta_{1}title\_dummy1_{i}\\ & +\beta_{2}title\_dummy2_{i}+\beta_{3}title\_dummy3_{i}+\beta_{4}title\_dummy4_{i}+\varepsilon_{i}{}^{\prime } \end{array}$$

#### 2.5.3. Model 3

The level of physicians’ education title may influence the decision of patients’ consultation behavior, so we added the education title as a *dummy* variable in model 3:

(8) $$\begin{array}{ll} \ln Patients_{i} & =\alpha_{0}+\alpha_{1}WeakTies_{i}+\alpha_{2}StrongTies_{i}+\alpha_{3}\left(WeakTies_{i}\times StrongTies_{i}\right)+\beta_{1}title\_dummy1_{i}\\ & +\ldots +\beta_{4}title\_dummy4_{i}+\chi_{1}edutitle\_dummy1_{i}+\ldots +\chi_{6}edutitle\_dummy6_{i}+\varepsilon_{i}{}^{\prime \prime } \end{array}$$

#### 2.5.4. Model 4

Information of outpatient, which is the highest level of outpatient consultation type that a physician can provide offline, may also influence the decision on patients’ consultation behavior, so we used outpatient information as another *dummy* variable in Model 4:

(9) $$\begin{array}{ll} \ln Patients_{i} & =\alpha_{0}+\alpha_{1}WeakTies_{i}+\alpha_{2}StrongTies_{i}+\alpha_{3}\left(WeakTies_{i}\times StrongTies_{i}\right)+\beta_{1}title\_dummy1_{i}\\ & +\ldots +\beta_{4}title\_dummy4_{i}+\chi_{1}edutitle\_dummy1_{i}+\ldots +\chi_{6}edutitle\_dummy6_{i}\\ & +\delta_{1}op\_dummy1_{i}+\ldots +\delta_{8}op\_dummy8_{i}+\varepsilon_{i}{}^{\prime \prime \prime } \end{array}$$

#### 2.5.5. Model 5

To explore the nature of the interaction between the influences of education title and strong ties, we introduced six interaction terms on the basis of Model 4:

(10) $$\begin{array}{ll} \ln Patients_{i} & =\alpha_{0}+\alpha_{1}WeakTies_{i}+\alpha_{2}StrongTies_{i}+\alpha_{3}\left(WeakTies_{i}\times StrongTies_{i}\right)+\beta_{1}title\_dummy1_{i}\\ & +\ldots +\beta_{4}title\_dummy4_{i}+\chi_{1}edutitle\_dummy1_{i}+\ldots +\chi_{6}edutitle\_dummy6_{i}\\ & +\delta_{1}op\_dummy1_{i}+\ldots +\delta_{8}op\_dummy8_{i}+\gamma_{1}strong\_edutitledummy1_{i}+\ldots \\ & +\gamma_{6}strong\_edutitledummy6_{i}+\varepsilon_{i}{}^{⁗} \end{array}$$

## 3. Results

The results are shown in Table 4. From experiments 1–5, it is seen that the coefficients for *WeakTies* and *StrongTies* are positive and significant, and the coefficients for *StrongWeakTies* are negative and significant. Therefore, hypotheses 1–3 are accepted. In addition, the coefficients for weak ties are on average 9.8 times stronger than the coefficients for strong ties.

It can be perceived that in Model 1, *WeakTies* has a positive and significant coefficient, suggesting that weak ties information positively affects patients’ consultation behavior $\left(\alpha_{1}=1528.062,\;p<0.01\right)$. *StrongTies* also has a positive and significant coefficient, suggesting that strong ties information positively affects patients’ consultation behavior $\left(\alpha_{2}=155.668,\;p<0.01\right)$. The coefficient of *StrongWeakTies* is negative and significant $\left(\alpha_{3}=-25,141.955,\;p<0.01\right)$, and $\;\alpha_{3}$ is larger than $\alpha_{1}\;$and $\alpha_{2}$. This is because the coefficients of *WeakTies* and *StrongTies* are between (0, 1), the value is smaller after multiplication, and the coefficient is greater in the regression. This result shows that in the online medical platform, the enhancement of weak ties information will reduce the positive effect of strong ties on the impact of physicians’ online consultations and vice versa.

In Model 2, the results for *WeakTies*, *StrongTies*, and *StrongWeakTies* are consistent with those of Model 1. Model 2 adds the title *dummy* variable. The coefficients for *StrongWeakTies* are negative and significant $(\alpha_{1}=1528.997,\;p<0.01;$ $\alpha_{2}=155.351,\;p<0.01;$ $\alpha_{3}=-25,099.596,\;p<0.01)$. The coefficients for chief physicians, attending physicians, and associate chief physicians are positive and significant $\left(\beta_{1}=0.596,\;p<0.01;\;\beta_{2}=0.696,\;p<0.01;\;\beta_{3}=0.680,\;p<0.01\right)$, while the coefficient for physicians was negative but not significant $\left(\beta_{4}=-0.105,\;p>0.1\right)$, indicating that the high title of physicians has a positive effect on patients’ consultation behavior.

Model 3 adds the *dummy* variable of education title to Model 2, the results for *WeakTies*, *StrongTies*, and *StrongWeakTies* are still consistent with those of Model 1 and Model 2, $\left(\alpha_{1}=1535.150,\;p<0.01;\;\alpha_{2}=155.315,\;p<0.01;\;\alpha_{3}=-25,122.476,\;p<0.01\right)$. The coefficients for chief physicians, attending physicians, and associate chief physicians are positive and significant $\left(\beta_{1}=0.649,\;p<0.01;\;\beta_{2}=0.615,\;p<0.01;\;\beta_{3}=0.661,\;p<0.01\right)$, and the coefficient for physicians is negative. In addition, the coefficient for no of information is small, positive, and significant $\left(\chi_{1}=0.094,\;p<0.01\right)$. The coefficients for associate Professor, associate Researcher, teaching Assistants, and lecturer are positive and significant $\left(\chi_{2}=0.080,\;p<0.01;\;\chi_{3}=0.401,\;p<0.1;\;\chi_{4}=0.269,\;p<0.1;\;\chi_{6}=0.510,\;p<0.01\right)$, indicating that education title plays a positive role in patients’ consultation behavior.

Model 4 adds the *dummy* variable, i.e., the highest level of outpatient consultation type that a physician can provide offline, to Model 3, and the results for *WeakTies*, *StrongTies*, and *StrongWeakTies* are still consistent with those of Model 1 $\left(\alpha_{1}=1474.437,\;p<0.01;\;\alpha_{2}=150.653,\;p<0.01;\;\alpha_{3}=-24,254.916,\;p<0.01\right)$; the coefficients for chief physicians, attending physicians, and associate chief physicians are positive and significant $\left(\beta_{1}=0.369,\;p<0.01;\;\beta_{2}=0.498,\;p<0.01;\;\beta_{3}=0.446,\;p<0.01\right)$; while coefficient for physicians was not significant $\left(\beta_{4}=-0.135,\;p>0.1\right)$ and performs the same with Model 3. The coefficients for the outpatient *dummy* are all positive and significant $(\delta_{1}=1.573,\;p<0.01;$ $\delta_{2}=0.428,\;p<0.01;$ $\delta_{3}=0.642,\;p<0.01;$ $\delta_{4}=0.541,\;p<0.01;$ $\delta_{5}=0.856,\;p<0.01;$ $\delta_{6}=0.725,\;p<0.01;$ $\delta_{7}=0.406,\;p<0.01;$ $\delta_{8}=0.795,\;p<0.01)$ and are bigger than the coefficients of the education title *dummy*. It means that outpatient type information has a positive impact on patients’ consultation behavior and is more important than physicians’ education title.

Model 5 added *StrongTies* to Model 4 with the cross-product term of *dummy* variable education title, and the positivity and significance of the main coefficients such as *WeakTies*, *StrongTies*, etc., remained unchanged $\left(\alpha_{1}=1475.074,\;\;p<0.01;\;\alpha_{2}=151.480,\;\;p<0.01;\;\alpha_{3}=-24,269.987,\;p<0.01\right)$. *StrongTies* had significant and negative coefficients with the two categories of teaching assistants and lecturers $\left(\;\gamma_{4}=-11.717,\;\;p<0.01;\;\gamma_{6}=-8.526,\;p<0.01\right)$, which conflicted with the sign of the coefficients for teaching assistants and lecturers $\left(\chi_{4}=0.364,\;p<0.05;\;\chi_{6}=0.585,\;p<0.01\right)$. Thus, *StrongTies* negatively adjusted for the role of education title when the education titles were teaching assistants and lecturers [13].

In Models 2 to 4, we add the *dummy* variables of title, education title, and outpatient type step by step. In addition, we add the cross term in Model 5. We also test $R^{2}$, *AIC*, and *BIC*. $R^{2}$ becomes bigger from 0.474 to 0.484, and *AIC* performs smaller from 173,591.7 to 172,810.8 in Models 1 to 5. *BIC* becomes smaller from 173,626.3 to 173,016.3 in Model 1 to 4 but becomes bigger in Model 5 (173,053). Considering these three tests, we think Model 5 performs the best, which indicates that education title information is useful to patients’ consultation behavior.

## 4. Robustness Test

We adopted the method of replacing samples for robustness testing and selected 52,645 homepage information of the same group of physicians on 25 January 2022 to construct the same model for validation.

As shown in the Table 5, all the conclusions are consistent with Models 1–5 in the original experiment. Only in Models 3–5 was the coefficient of education title *dummy* of associate researcher performs significant, which is the same as other education title and does not affect the conclusion of the original variable analysis.

## 5. Discussion

Through the above experiments, we can see that both strong and weak ties information has a positive effect on patients’ decision of whether to choose the physician for consultation [14]. The information displayed on a physician’s homepage on the online medical website helps patients to judge the physician’s professional competence and service attitude and thus make a physician consultant decision.

Furthermore, we found that weak ties information is, on average, 9.8 times more influential than strong ties information in comparison to the influence on patients’ consultation behavior. Therefore, we can conclude that strong ties information does not have a strong positive relationship with patients’ physician consultation behavior. Instead, weak ties information has a greater positive impact on patients’ consultation behavior. That is, it is not the case that the stronger the interaction between the physician and the patient, the greater the positive effect of the generated information on the patient’s choice of the physician for consultation. In other words, the more intuitive information, that is, weak tie information, displayed on the physician’s homepage without deep interaction between the physician and the patient, the stronger the positive effect on patients’ consultation behavior. This also validates the findings of Granovetter (1973) [25], whose study concluded that weak interpersonal relationships are more important in the process of information dissemination than strong relationships. In addition, it is clear that if we change the initial variables, their influence on dependent variables will also be changed, which can be seen from the coefficients. However, the correlativity between weak and strong ties information will not change. For example, the coefficient of “*WeakTies*” is always bigger than the coefficient of “*StrongTies*”.

On the other hand, this also shows that it is necessary for physicians to take some actions, such as proactively generating objective data, adding more expertise, etc., to maintain their homepage, so as to facilitate patients to understand their state of disease and judge the physician’s professionalism. For example, patients can obtain a deeper understanding of a physician’s knowledge of a certain disease from articles published on Health subscription, which is more effective than the information about the physician’s interaction with the patient.

We also found that even though both strong and weak ties information have a positive effect on patients’ consultation behavior, the enhancement of one side’s information will reduce the positive effect of the other side’s information on the consultation service. Strong ties information can certainly show the physician’s high quality consultation service and patient’s recognition, but combined with the conclusion that weak ties information is on average 9.8 times more influential than strong ties information, we suggest that physicians need to pay more attention to the role of weak ties information. Strong ties information is more concerned with the good results brought by the quality of consultation service, while weak ties information should be regarded as the focus of personal brand maintenance, so as to increase the number of consultations most efficiently.

However, in the exploration of *dummy* variables, we found that information such as title, education title, and outpatient type can impact the choice of patients slightly. In addition, the outpatient type and title information are more important than the education title. They all have positive impacts on patients’ consultation behavior.

In this paper, we innovatively study the patients’ consultation behavior from the perspective of social ties and explore the influence of strong and weak ties information on it. However, our study still has certain limitations. First, our data sources are limited, and we only crawled the data of Online Medical Platform A, a well-known Chinese online healthcare platform. However, there are important differences across the different media [30], and big data for health services are now becoming very popular [35]. As a result, whether the findings are applicable to all online healthcare platforms needs to be discussed in further studies. Second, the *dummy* variables we included in the model were all considered to show the objective professionalism of physicians. We explore the influence of title, education title, and the highest level of offline outpatient types on patients’ consultation behavior, but the exploration of other information about physicians is insufficient. Third, we may consider the effect of physicians’ group performance in the future [36].

In summary, our research provides support for further exploration of the role of strong and weak ties information on users in the online healthcare field. Future research could further explore how strong and weak ties affect other patient behaviors. It could also further mine textual information to explore the impact of online social text information on patient behavior.

## 6. Conclusions

The following conclusions and suggestions can be drawn from this paper:

Both strong and weak ties have a positive effect on patients’ consultation behavior. However, the enhancement of informativeness on one side will reduce the positive effect of information on the other side on the patients’ consultation behavior.

In comparison of the impact on the patients’ consultation behavior, weak ties information was on average 9.8 times more influential than strong ties information. Weak ties variables have a more positive effect on patients’ consultation decision.

Based on the first and second conclusions, this paper suggests that physicians should pay more attention to improving the quality of weak ties information, to efficiently attract more patients and increase the number of consultations.

Information such as the physician ‘s title, education level and the highest type of outpatient physicians can offer offline, which can represent the physician ‘s professional competence, can have a positive impact on patients’ physician consultation decision. The patients’ consultation behavior can reflect patients’ recognition of the physician. Therefore, this paper suggests that physicians should strive to improve their professional competence and obtain a high professional title and to increase patients’ recognition and trust.

## Figures and Tables

**Table 1:** Definitions and descriptive statistics of all original variables.

			(1)	(2)	(3)	(4)	(5)
Variables	Definition	Type	N	Mean	Sd	Min	Max
Number of checked patients (*NCP*)	The number of patients who scanned the physician’s QR code and checked in on Online Medical Platform A after the offline consultation.	Strong ties variable	42,319	267.6	1063	0	44,032
Number of comments after consultation (*NCC*)	The number of comments made by the patient to the physician after the consultation.	Strong ties variable	42,319	56.51	175.2	0	4481
Number of thank-you letters (*NTL*)	The number of thank-you letters received online by physicians.	Strong ties variable	42,319	22.93	78.68	0	2185
Number of gifts (*NOG*)	The number of gifts received online by physicians.	Strong ties variable	42,319	54.96	230.2	0	9788
Number of followers (*NOF*)	The number of followers on the physician’s website Health subscription.	Strong ties variable	42,319	501.4	1660	0	52,000
Comprehensive recommendation score (*CRS*)	Comprehensive recommendation score rated by Online Medical Platform A	Weak ties variable	42,319	3.266	0.395	1.700	5
Number of articles on Health subscription (*NAH*)	The number of articles published by physicians on the physicians’ website Health subscription.	Weak ties variable	42,319	17.33	462.8	0	93,047
Number of articles reads (*NAR*)	The number of reads of articles published on the physician’s website Health subscription.	Weak ties variable	42,319	69,424	977,273	0	1.819 × 10^8^
Total number of visits (*TNV*)	The total number of visits to the physicians’ homepage.	None	42,319	867,222	3.744 × 10^6^	1000	3.001 × 10^8^
Patients	Total number of patients who have consulted physicians.	Dependent variable	42,319	885.0	2808	1	127,595

**Table 2:** Definitions of the Variables and summary statistics.

			(1)	(2)	(3)	(4)	(5)
Variables	Definition	Type	N	Mean	Sd	Min	Max
*WeakTies*	Weak ties index	Interval	42,319	0.00269	0.000918	0.00144	0.00715
*StrongTies*	Strong ties index	Interval	42,319	0.00781	0.0210	0	0.139
*StrongWeakTIes*	Interaction of weak ties and strong ties	Interval	42,319	3.48 × 10^5^	0.000116	0	0.000838
*patients*	The min-maxed total number of patients who have consulted physicians.	Interval	42,319	−7.579	2.593	−11.76	−2.304
*title*_*dummy*1	*title*_*dummy*1 = 1 if title = “Chief Physician”, but zero otherwise.	*Dummy*	42,319	0.552	0.497	0	1
*title*_*dummy*2	*title*_*dummy*2 = 1 if title = “Attending Physician”, but zero otherwise.	*Dummy*	42,319	0.0904	0.287	0	1
*title*_*dummy*3	*title*_*dummy*3 = 1 if title = “Associate Chief Physician”, but zero otherwise.	*Dummy*	42,319	0.339	0.473	0	1
*title*_*dummy*4	*title*_*dummy*4 = 1 if title = “Physician”, but zero otherwise.	*Dummy*	42,319	0.0116	0.107	0	1
*edutitle*_*dummy*1	*edutitle*_*dummy*1 = 1 if title = “None”, but zero otherwise.	*Dummy*	42,319	0.480	0.500	0	1
*edutitle*_*dummy*2	*edutitle*_*dummy*2 = 1 if title = “Associate Professor”, but zero otherwise.	*Dummy*	42,319	0.199	0.399	0	1
*edutitle*_*dummy*3	*edutitle*_*dummy*3 = 1 if title = “Associate Researcher”, but zero otherwise.	*Dummy*	42,319	0.00142	0.0376	0	1
*edutitle*_*dummy*4	*edutitle*_*dummy*4 = 1 if title = “Teaching Assistants”, but zero otherwise.	*Dummy*	42,319	0.00359	0.0598	0	1
*edutitle*_*dummy*5	*edutitle*_*dummy*5 = 1 if title = “Researcher”, but zero otherwise.	*Dummy*	42,319	0.00376	0.0612	0	1
*edutitle*_*dummy*6	*edutitle*_*dummy*6 = 1 if title = “Lecturer”, but zero otherwise.	*Dummy*	42,319	0.0567	0.231	0	1
*op*_*dummy*1	*op*_*dummy*1 = 1 if physicians’ highest level of outpatient = “VIP”, but zero otherwise.	*Dummy*	42,319	0.000425	0.0206	0	1
*op*_*dummy*2	*op*_*dummy*2 = 1 if physicians’ highest level of outpatient = “Experts”, but zero otherwise.	*Dummy*	42,319	0.576	0.494	0	1
*op*_*dummy*3	*op*_*dummy*3 = 1 if physicians’ highest level of outpatient = “Specialized medical outpatient”, but zero otherwise.	*Dummy*	42,319	0.0255	0.158	0	1
*op*_*dummy*4	*op*_*dummy*4 = 1 if physicians’ highest level of outpatient = “other”, but zero otherwise.	*Dummy*	42,319	0.00340	0.0582	0	1
*op*_*dummy*5	*op*_*dummy*5 = 1 if physicians’ highest level of outpatient = “Famous”, but zero otherwise.	*Dummy*	42,319	0.00463	0.0679	0	1
*op*_*dummy*6	*op*_*dummy*6 = 1 if physicians’ highest level of outpatient = “International”, but zero otherwise.	*Dummy*	42,319	0.0127	0.112	0	1
*op*_*dummy*7	*op*_*dummy*7 = 1 if physicians’ highest level of outpatient = “General”, but zero otherwise.	*Dummy*	42,319	0.0636	0.244	0	1
*op*_*dummy*8	*op*_*dummy*8 = 1 if physicians’ highest level of outpatient = “Special needed”, but zero otherwise.	*Dummy*	42,319	0.137	0.344	0	1

**Table 3:** Correlations of the Variables.

**(obs = 42,319)**									
	*patients*	*WeakTies*	*Strong*~*s*	*Strongw*~*s*	*title*~1	*titl*~2	*title*~3	*title*~4	*edutit*~1
*patients*	1								
*WeakTies*	0.595	1							
*StrongTies*	0.547	0.691	1						
*StrongWeakTies*	0.477	0.724	0.963	1					
*title dummy*1	0.121	0.211	0.0574	0.0483	1				
*title dummy*2	−0.0873	−0.148	−0.0465	−0.0377	−0.350	1			
*title dummy*3	−0.0451	−0.105	−0.0211	−0.0194	−0.795	−0.226	1		
*title dummy*4	−0.0922	−0.0933	−0.0342	−0.0281	−0.120	−0.0342	−0.0777	1	
*edutitle d*~1	−0.182	−0.277	−0.103	−0.0902	−0.265	0.164	0.155	0.0801	1
*edutitle d*~2	0.0646	0.0950	0.0388	0.0324	−0.0852	−0.150	0.197	−0.0540	−0.479
*edutitle d*~3	0.0123	0.0127	0.00360	0.00250	−0.0267	0.00780	0.0168	0.00180	−0.0362
*edutitle d*~4	−0.0196	−0.0314	−0.00710	−0.00660	−0.0627	0.0637	−0.00880	0.141	−0.0577
*edutitle d*~5	0.0128	0.0151	0.00680	0.00570	−0.00680	0.0278	−0.0106	−0.00310	−0.0591
*edutitle d*~6	0.0238	−0.0284	0.00110	0.000800	−0.226	0.226	0.100	0.00390	−0.236
*op dummy*1	0.0153	0.00970	0.00970	0.00970	0.0117	−0.00650	−0.00750	−0.00220	0.00540
*op dummy*2	−0.0131	−0.00730	−0.0523	−0.0477	0.148	−0.328	0.0833	−0.124	−0.0676
*op dummy*3	0.0249	−0.00390	0.00750	0.00270	−0.107	0.151	0.0216	−0.000800	0.0278
*op dummy*4	0.0178	0.0198	0.0139	0.0119	−0.00940	0.00850	0.00440	−0.00260	−0.00670
*op dummy*5	0.0339	0.0342	0.0247	0.0223	0.0502	−0.0215	−0.0371	−0.00740	−0.000800
*op dummy*6	0.0780	0.0924	0.0615	0.0521	0.0348	−0.0247	−0.0176	−0.0123	−0.0644
*op dummy*7	−0.0584	−0.110	−0.0472	−0.0403	−0.258	0.418	−0.00310	0.0747	0.106
*op dummy*8	0.232	0.250	0.190	0.166	0.224	−0.119	−0.149	−0.0407	−0.116
*strong edu*~1	0.302	0.355	0.547	0.520	−0.0309	0.00180	0.0366	−0.0179	0.221
*strong edu*~2	0.253	0.328	0.475	0.460	−0.0359	−0.0520	0.0757	−0.0185	−0.164
*strong edu*~3	0.0247	0.0265	0.0354	0.0339	−0.0138	0.0130	0.00530	−0.00170	−0.0154
*strong edu*~4	0.0256	0.0277	0.0518	0.0448	−0.0175	0.0138	0.00680	0.0153	−0.0152
*strong edu*~5	0.0377	0.0466	0.0680	0.0642	0.00120	−0.00470	−0.000800	−0.00260	−0.0227
*strong edu*~6	0.124	0.154	0.229	0.218	−0.0774	0.0766	0.0352	−0.000600	−0.0796
	edutit~2	edutit~3	edutit~4	edutit~5	edutit~6	op dum~1	op dum~2	op dum~3	op dum~4
*edutitle d*~2	1								
*edutitle d*~3	−0.0188	1							
*edutitle d*~4	−0.0299	−0.00230	1						
*edutitle d*~5	−0.0306	−0.00230	−0.00370	1					
*edutitle d*~6	−0.122	−0.00920	−0.0147	−0.0151	1				
*op dummy*1	0.00120	−0.000800	−0.00120	−0.00130	−0.00510	1			
*op dummy*2	0.112	−0.00700	−0.0443	−0.0129	−0.0781	−0.0240	1		
*op dummy*3	−0.0134	0.00190	0.0128	0.00230	0.0749	−0.00330	−0.189	1	
*op dummy*4	−0.00470	−0.00220	0.0101	−0.00360	0.0138	−0.00120	−0.0680	−0.00950	1
*op dummy*5	−0.0183	−0.00260	−0.00410	−0.00420	−0.0107	−0.00140	−0.0794	−0.0110	−0.00400
*op dummy*6	0.0180	0.00130	−0.00680	−0.00350	−0.0160	−0.00230	−0.132	−0.0184	−0.00660
*op dummy*7	−0.0819	0.00820	0.0475	0.0141	0.165	−0.00540	−0.303	−0.0422	−0.0152
*op dummy*8	−0.0203	0.00140	−0.0239	0.00690	−0.0734	−0.00820	−0.465	−0.0646	−0.0233
*strong edu*~1	−0.106	−0.00800	−0.0127	−0.0130	−0.0520	0.0130	−0.0247	0.0157	0.00860
*strong edu*~2	0.343	−0.00640	−0.0103	−0.0105	−0.0419	0.00730	0.00240	−0.00540	0.00120
*strong edu*~3	−0.00800	0.425	−0.00100	−0.00100	−0.00390	−0.000300	−0.00430	0.000400	−0.000900
*strong edu*~4	−0.00790	−0.000600	0.264	−0.00100	−0.00390	−0.000300	−0.00120	0.0189	−0.000900
*strong edu*~5	−0.0118	−0.000900	−0.00140	0.385	−0.00580	−0.000500	−0.000100	−0.00270	−0.00140
*strong edu*~6	−0.0412	−0.00310	−0.00500	−0.00510	0.338	−0.00170	−0.0222	0.0564	0.0193
	op dum~5	op dum~6	op dum~7	op dum~8	strong~1	strong~2	strong~3	strong~4	strong~5
*op dummy*5	1								
*op dummy*6	−0.00770	1							
*op dummy*7	−0.0178	−0.0296	1						
*op dummy*8	−0.0272	−0.0453	−0.104	1					
*strong edu*~1	0.0268	0.00450	−0.00570	0.0800	1				
*strong edu*~2	0.00720	0.0287	−0.0381	0.0622	−0.0363	1			
*strong edu*~3	−0.00110	−0.00140	0.0129	0.00230	−0.00340	−0.00270	1		
*strong edu*~4	−0.00110	−0.00180	0.0106	−0.00630	−0.00340	−0.00270	−0.000300	1	
*strong edu*~5	−0.00160	−0.00120	0.000600	0.0111	−0.00500	−0.00400	−0.000400	−0.000400	1
*strong edu*~6	−0.00540	−0.00100	0.0266	−0.00180	−0.0176	−0.0141	−0.00130	−0.00130	−0.00200
	strong~6								
*strong edu*~6	1								

**Table 4:** Results.

	(1)	(2)	(3)	(4)	(5)
Variables	y	y	y	y	y
*WeakTies*	1528.062 ***	1528.997 ***	1535.150 ***	1474.437 ***	1475.074 ***
	(101.12)	(95.74)	(91.96)	(89.15)	(88.41)
*StrongTies*	155.668 ***	155.351 ***	155.315 ***	150.653 ***	151.480 ***
	(34.13)	(33.99)	(33.99)	(33.36)	(32.78)
*StrongWeakTies*	−25,141.955 ***	−25,099.596 ***	−25,122.476 ***	−24,254.916 ***	−24,269.987 ***
	(−28.80)	(−28.60)	(−28.57)	(−27.92)	(−27.78)
*title*_*dummy*1		0.596 ***	0.649 ***	0.369 ***	0.365 ***
		(4.64)	(5.05)	(2.91)	(2.87)
*title*_*dummy*2		0.696 ***	0.615 ***	0.498 ***	0.498 ***
		(5.31)	(4.68)	(3.86)	(3.84)
*title*_*dummy*3		0.680 ***	0.661 ***	0.446 ***	0.443 ***
		(5.30)	(5.15)	(3.53)	(3.50)
*title*_*dummy*4		−0.105	−0.130	−0.135	−0.135
		(−0.70)	(−0.86)	(−0.91)	(−0.90)
*edutitle*_*dummy*1			0.094 ***	0.110 ***	0.110 ***
			(3.51)	(4.15)	(3.84)
*edutitle*_*dummy*2			0.080 ***	0.090 ***	0.106 ***
			(2.74)	(3.10)	(3.34)
*edutitle*_*dummy*3			0.401 *	0.393	0.318
			(1.65)	(1.61)	(1.15)
*edutitle*_*dummy*4			0.269 *	0.300 *	0.364 **
			(1.66)	(1.84)	(2.13)
*edutitle*_*dummy*5			0.205	0.211	0.247
			(1.41)	(1.47)	(1.54)
*edutitle*_*dummy*6			0.510 ***	0.517 ***	0.585 ***
			(10.99)	(11.12)	(11.86)
*op*_*dummy*1				1.573 ***	1.567 ***
				(4.33)	(4.26)
*op*_*dummy*2				0.428 ***	0.429 ***
				(14.88)	(14.92)
*op*_*dummy*3				0.642 ***	0.649 ***
				(10.90)	(11.07)
*op*_*dummy*4				0.541 ***	0.547***
				(3.65)	(3.70)
*op*_*dummy*5				0.856 ***	0.850 ***
				(6.70)	(6.63)
*op*_*dummy*6				0.725 ***	0.721 ***
				(9.93)	(9.88)
*op*_*dummy*7				0.406 ***	0.401 ***
				(8.98)	(8.87)
*op*_*dummy*8				0.795 ***	0.792 ***
				(21.83)	(21.75)
*strong*_*edutitledummy*1					0.590
					(0.56)
*strong*_*edutitledummy*2					−1.660
					(−1.58)
*strong*_*edutitledummy*3					7.585
					(1.11)
*strong*_*edutitledummy*4					−11.717 ***
					(−3.41)
*strong*_*edutitledummy*5					−3.635
					(−1.02)
*strong*_*edutitledummy*6					−8.526 ***
					(−5.04)
*Constant*	−12.031 ***	−12.654 ***	−12.777 ***	−12.794 ***	−12.799 ***
	(−299.68)	(−96.85)	(−95.09)	(−97.18)	(−96.36)
*Observations*	42,319	42,319	42,319	42,319	42,319
*R-squared*	0.474	0.475	0.477	0.484	0.484
*AIC*	173,591.7	173,470.9	173,360.1	172,825.9	172,810.8
*BIC*	173,626.3	173,540.1	173,481.2	173,016.3	173,053

Robust *t*-statistics in parentheses, *** *p* < 0.01, ** *p* < 0.05, * *p* < 0.1.

**Table 5:** Robustness test.

	(1)	(2)	(3)	(4)	(5)
Variables	y	y	y	y	y
*WeakTies*	1569.418 ***	1569.663 ***	1575.592 ***	1517.411 ***	1517.953 ***
	(100.34)	(95.00)	(91.29)	(88.77)	(88.06)
*StrongTies*	154.751 ***	154.428 ***	154.366 ***	150.006 ***	150.806 ***
	(34.46)	(34.31)	(34.30)	(33.68)	(33.09)
*StrongWeakTies*	−25,721.187 ***	−25,673.125 ***	−25,690.949 ***	−24,861.893 ***	−24,877.057 ***
	(−29.07)	(−28.86)	(−28.82)	(−28.19)	(−28.06)
*title*_*dummy*1		0.592 ***	0.645 ***	0.378 ***	0.373 ***
		(4.60)	(5.01)	(2.97)	(2.92)
*title*_*dummy*2		0.686 ***	0.605 ***	0.495 ***	0.494 ***
		(5.22)	(4.60)	(3.82)	(3.80)
*title*_*dummy*3		0.675 ***	0.656 ***	0.452 ***	0.449 ***
		(5.26)	(5.10)	(3.57)	(3.53)
*title*_*dummy*4		−0.115	−0.140	−0.147	−0.146
		(−0.76)	(−0.92)	(−0.98)	(−0.97)
*edutitle*_*dummy*1			0.092 ***	0.108 ***	0.107 ***
			(3.46)	(4.07)	(3.74)
*edutitle*_*dummy*2			0.079 ***	0.090 ***	0.105 ***
			(2.73)	(3.10)	(3.33)
*edutitle*_*dummy*3			0.392	0.381	0.306
			(1.61)	(1.56)	(1.10)
*edutitle*_*dummy*4			0.293 *	0.326 **	0.387 **
			(1.81)	(2.01)	(2.28)
*edutitle*_*dummy*5			0.199	0.205	0.237
			(1.37)	(1.43)	(1.48)
*edutitle*_*dummy*6			0.511 ***	0.518 ***	0.587 ***
			(11.01)	(11.14)	(11.91)
*op*_*dummy*1				1.552 ***	1.546 ***
				(4.25)	(4.18)
*op*_*dummy*2				0.405 ***	0.406 ***
				(14.28)	(14.30)
*op*_*dummy*3				0.625 ***	0.632 ***
				(10.70)	(10.87)
*op*_*dummy*4				0.453 **	0.446 **
				(2.40)	(2.35)
*op*_*dummy*5				0.832 ***	0.826 ***
				(6.51)	(6.44)
*op*_*dummy*6				0.715 ***	0.711 ***
				(9.87)	(9.81)
*op*_*dummy*7				0.383 ***	0.377 ***
				(8.48)	(8.36)
*op*_*dummy*8				0.766 ***	0.763 ***
				(21.24)	(21.16)
*strong*_*edutitledummy*1					0.682
					(0.65)
*strong*_*edutitledummy*2					−1.651
					(−1.57)
*strong*_*edutitledummy*3					7.567
					(1.12)
*strong*_*edutitledummy*4					−11.027 ***
					(−3.21)
*strong*_*edutitledummy*5					−3.236
					(−0.86)
*strong*_*edutitledummy*6					−8.676 ***
					(−5.15)
*Constant*	−12.006 ***	−12.622 ***	−12.744 ***	−12.761 ***	−12.766 ***
	(−298.88)	(−96.36)	(−94.62)	(−96.54)	(−95.73)
*Observations*	42,270	42,270	42,270	42,270	42,270
*R-squared*	0.474	0.476	0.477	0.484	0.484
*AIC*	173353	173233.1	173121.6	172620.5	172604.4
*BIC*	173387.6	173302.3	173242.8	172810.8	172846.6

Robust *t*-statistics in parentheses, *** *p* < 0.01, ** *p* < 0.05, * *p* < 0.1.

## Data Availability

The image data used to support the findings of this study are available from the corresponding author upon request. The platform where the information is collected has been named by a particular name: Online Medical Platform A. All data have been desensitized, and there is no content that has privacy issues for any of the related parties.
